# Epidemiology of Intracranial Meningiomas in Mexico: Cohort of the National Institute of Neurology and Neurosurgery During the Last Decade

**DOI:** 10.7759/cureus.40046

**Published:** 2023-06-06

**Authors:** Juan Antonio Alvaro-Heredia, Noe Alejandro Salazar Felix, German López-Valencia, Tomas Moncada-Habib, Jorge Ivan Castro-Vega, Luis A Rodríguez-Hernández, Michel Mondragón-Soto, Marco Antonio Muñuzuri-Camacho, Isidro Alvaro-Heredia, Alberto González-Aguilar

**Affiliations:** 1 Neurosurgery, Instituto Nacional de Neurología y Neurocirugía Manuel Velasco Suárez, Mexico City, MEX

**Keywords:** world health organization (who), supratentoraial meningioma, latin american, epidemiology and biostatistics, primary brain tumors

## Abstract

Introduction

Meningiomas have been described as slow-growing neoplasms with benign behavior derived from the connective tissue surrounding the brain and spinal cord. Meningiomas represent one-third of primary central nervous system (CNS) tumors. The World Health Organization (WHO) initially classified them into three groups based on their histopathological characteristics, recently incorporating molecular patterns. Small cohorts have been reported in Latin America compared to the international literature. Ignoring the epidemiology of meningiomas in this region and considering this limitation, we aim to study the epidemiology of meningiomas in our country, Mexico.

Material and methods

A historical cohort was carried out on 916 patients diagnosed with intracranial meningiomas from January 2008 to January 2021, considering sociodemographic, topographic, and histopathological characteristics.

Results

In this study, 69.4% (n=636) of patients were women with a mean overall age of 47.53 (SD=14.85) years; 79.6% (n=729) of the lesions were supratentorial with convexity meningiomas being the most prevalent at 32.6% (n=299). Histopathologically, transitional (45.7%) (n=419), meningothelial (22.1%) (n=202), and fibroblastic (16.7%) (n=153) meningiomas were the most frequent. We found significant differences between men and women in age (p=0.01), infra or supratentorial presentation (p<0.001), location of the lesion (p<0.001), and histopathological characteristics (p<0.001).

Conclusions

Our results are consistent with what has been reported; however, until now, it appears as the largest series reported in our country and Latin America.

## Introduction

Meningiomas are the most common tumors in the central nervous system, representing between 26-34% of tumors [1]. Meningiomas are neoplasms originating from cap cells of the arachnoid and are benign lesions, and however, on some occasions, they can present an aggressive behavior depending on their histopathological grade [2-4]. They are classified into three grades according to the histopathological parameters established by the World Health Organization; fortunately, more than 80% of these lesions are grade 1, 16.9% are grade 2, and only 1.7% are grade 3 [3,4]. Meningiomas are more common in people older than 65 years, predominantly in women, probably related to the level of sex hormones in women [1,5,6]. The clinical presentation depends on the location of the lesion and is related to the compression of neurovascular structures [8]; according to those described by Al-Mefty, the vast majority are supratentorial, of which the most frequent are those of convexity [8-10].

Despite how common these lesions are, epidemiological studies are poor compared to other tumors such as gliomas [11], few studies describe the clinical-epidemiological characteristics of these lesions, and the few available are described in different populations [12-16]. In Latin America, very few studies describe the clinical-epidemiological characteristics of these lesions, so it is vital to promote, update, and contribute studies that help enrich the knowledge of these lesions to have a greater scope in terms of public health and better statistics in our country and Latin America [17-19].

## Materials and methods

A retrospective cohort descriptive study was conducted. Consecutive patients of all ages attending the Neurosurgery Department at the National Institute of Neurology and Neurosurgery in Mexico City between January 2008 to January 2021 were included.

All individuals with a brain MRI suggestive of meningioma confirmed by histopathology were included. Patients with tumors of other etiologies and/or a negative histopathology diagnosis were excluded. Information was sought within the physical and electronic records.

The demographic variables registered included gender and age in ranges of 0-20y, 21-40y, 41-60y, and >60y. Tumors were dichotomously categorized as supratentorial or infratentorial lesions. Location was described according to the MRI radiographic features, including lesions in the convexity, cerebellopontine angle, sellar and parasellar, parasagittal and falx, skull base, medullary, intraventricular, and foramen magnum. Categorization by subtypes was defined according to the WHO classification of CNS tumors, being recognized and divided as angiomatous, atypical, anaplastic, chordoid, clear cell, fibrous, lymphoplasmacytic-rich, meningothelial, metaplastic, microcystic, papillary, psammomatous, rhabdoid, secretory, and transitional. Classification between grades was defined according to the WHO classification of CNS tumors, which ranks 1 to 3 based on histological and molecular characteristics.

For the statistical analysis, quantitative variables were described with mean and standard deviation (SD). Qualitative variables were described with frequencies and percentages. Chi-square tests were conducted to identify significant differences between groups. A p-value of <0.05 was used for statistical significance. Statistical analyses were performed using SPSS 25 (IBM).

All included patients who agreed to participate signed the informed consent. The local Ethics Committee approved the study.

## Results

A total of 916 patients (69.4% female) with a mean age of 47.53±14.85 years (y) were included. Subjects between 41-60y were more prevalent, with 428 individuals (46.7%). According to location, supratentorial lesions include 729 patients (79.6%), convexity injuries enclose 299 patients (32.6%), followed by 199 who had skull base (21.7%), and parasagittal were documented in 168 individuals (18.3). WHO grade 1 histopathological finding were represented in 814 individuals (88.9%). Regarding the histopathological spectrum, transitional meningiomas were reported in 419 tissues (45.7%), following meningothelial in 202 (22.1%), and fibrous in 153 specimens (16.7%). Table 1 shows the complete description of the patients.

**Table 1:** Description of the patients. Description of the general characteristics of the complete sample. SD - Standard Deviation

Description	
Female, n (%)	636 (69.4)
Age, mean (SD)	47.5 (±14.8)
0-20y, n (%)	39 (4.3)
21-40y, n (%)	256 (27.9)
41-60y, n (%)	428 (46.7)
>60y, n (%)	193 (21.1)
Location	
Supratentorial, n (%)	729 (79.6)
Infratentorial, n (%)	187 (20.4)
Source	
Foramen magnum, n (%)	10 (1.1)
Skull base, n (%)	199 (21.7)
Convexity, n (%)	299 (32.6)
Intraventricular, n (%)	20 (2.2)
Medular, n (%)	77 (8.4)
Parasagittal, n (%)	168 (18.3)
Cerebellopontine angle, n (%)	92 (10)
Sellar/Parasellar, n (%)	51 (5.6)
Malignancy	
Grade 1, n (%)	814 (88.9)
Grade 2, n (%)	9 (1)
Grade 3, n (%)	93 (10.2)
Histopathological subtype	
Meningothelial, n (%)	202 (22.1)
Fibrous, n (%)	153 (16.7)
Transitional, n (%)	419 (45.7)
Psammomatous, n (%)	35 (3.8)
Angiomatous, n (%)	3 (0.3)
Metaplastic, n (%)	2 (0.2)
Atypical, n (%)	5 (0.5)
Chordoid, n (%)	2 (0.2)
Clear cell, n (%)	2 (0.2)
Papillary, n (%)	77 (8.4)
Anaplastic, n (%)	16 (1.7)

In the comparative analysis, performing by sex, statistical significance was found in age of presentation (p=0.001), infra or supratentorial classification (p<0.001), lesion topography (p<0.001), and histopathological spectrum (p<0.001). Table 2 shows a full comparative analysis between sex.

**Table 2:** Full comparative analysis Comparison between sex. (‡) Chi-Squared Test

Characteristics	Female, n=636	Male, n=280	p
Age			0.001 ‡
0-20y, n	21	18	
21-40y, n	159	97	
41-60y, n	314	114	
>60y, n	142	51	
Location			<0.001 ‡
Supratentorial, n (%)	481	155	
Infratentorial, n (%)	248	32	
Source			<0.001 ‡
Convexity, n (%)	188	111	
Skull base, n (%)	147	52	
Foramen magnum, n (%)	9	1	
Intraventricular, n (%)	12	8	
Medular, n (%)	60	17	
Parasagittal, n (%)	100	68	
Cerebellopontine angle, n (%)	79	13	
Sellar/Parasellar, n (%)	41	10	

## Discussion

In recent decades, an increase in the incidence of central nervous system tumors has been observed, particularly in meningiomas. According to the statistics established by CBTR [5], these are the most commonly reported tumors. Despite the importance of this diagnosis, epidemiological studies in Latin America are scarce. In Mexico, Anaya et al. [20] describe meningiomas as the most common pathology, with a percentage of 22.8% in a series of 9615 patients. Currently, only one study in the Mexican population has evaluated meningiomas; this information is available from a cohort study by Ancer et al., which included only 167 patients, with results that are not very representative [19].

Regarding the demographic characteristics of our study, we found that the mean age of presentation was 47.5 ±14.85 years, slightly below those described in other series where it is more common over 65 years [5-7].

Regarding the differences between the sexes, there is a marked predisposition for the female sex [21], for more than a century, Cushing established that this is due in part to the hormonal influence of the female sex due to the presence of progesterone receptors [11,21,22]. In our study, the female preponderance agrees with the information published in other populations, of 916 included patients, 69.4% were female.

Ketter et al. describe the most common location in a series of 661 cases, describe that those of the convexity and base of the skull are the most frequent, with percentages of 58% and 30%, respectively [23]; the results of our study agree with In the literature, we found that convexity meningiomas correspond to 32.6% of the cases, those of the base of the skull to 21.7% and the parasagittal to 18.3%.

According to the data reported in the Central Brain Tumor Registry of the United States (CBTRUS), histopathological, grade 1 tumors correspond to 80% [5], and our study reported 88.9% of grade 1 lesions, reinforcing the statistics presented by other populations. On the other hand, grade 2 lesions correspond to 5%-17% in the international literature [24], and our study reported 1%; these results are different from what has been reported in the literature; we suggest that the delay in diagnosis and the lack of resources to identify genetic mutations, such as mutations of the Telomerase reverse transcriptase (TERT) promoter, in developing countries like ours are involved in malignant progression and misdiagnosis of a grade 2 meningioma [18] Grade 3 lesions constitute 1-3% of all meningiomas [25]; in our series, we found 10% of these lesions.

In the comparative analysis, performing by sex, statistical significance was found in the age of presentation (p=0.01), infra or supratentorial classification (p<0.001), injury location (p<0.001), and histopathological spectrum (p<0.001).

This study's strengths lies in the Mexican population's cohort size. To our knowledge, this study represents the biggest series in Latin America. However, this study is not free from limitations; due to the retrospective nature of our analysis and the WHO criteria changes during the study period, the molecular characterization of meningiomas was not possible. This characterization may open the door to better approaches with the hope of improved patient outcomes. Further studies are needed to investigate these mutational profiles in the Latin population.

## Conclusions

Despite how common these lesions are, epidemiological studies are scarce compared to other tumors, such as gliomas. Few studies describe the clinical-epidemiological characteristics of these lesions, and the few available are described in different populations. In Latin America, there are very few studies that describe the clinical-epidemiological characteristics of these tumors, so it is of vital importance to promote, update, and contribute studies that help to enrich the knowledge of these lesions, to have a greater scope in terms of public health as well as better statistics in our country and Latin America. In the future, further studies will be necessary to incorporate molecular variables into the histopathological findings in our country.
